# Panoramic Dominance of the Immune System in Cardiorenal Syndrome Type I

**DOI:** 10.7759/cureus.9869

**Published:** 2020-08-19

**Authors:** Venkata Sri Ramani Peesapati, Mohammad Sadik, Sadhika Verma, Marline A Attallah, Safeera Khan

**Affiliations:** 1 Research, California Institute of Behavorial Neurosciences and Psychology, Fairfield, USA; 2 Internal Medicine, Manipal College of Medical Sciences, Pokhara, NPL; 3 Internal Medicine, California Institute of Behavorial Neurosciences and Psychology, Fairfield, USA

**Keywords:** cardiorenal syndrome type i, inflammation, acute kidney injury, cytokines, toll-like receptors, innate immune system, apoptosis, oxidative stress

## Abstract

Physiological organ cross-talk is necessary to maintain equilibrium and homeostasis. Heart and kidney are the essences of this equilibrium. Organ failure in either of these organs can perturb the bidirectional communication between them, impinging this unpleasant vascular and cellular milieu on other distant organs. Cardiorenal syndrome (CRS) type I occurs due to acute deterioration of cardiac function, ultimately causing acute kidney injury (AKI). This syndrome is an intricate condition with neurohormonal and inflammatory aspects. Inflammation creates a vicious circle filled with the innate and adaptive immune systems, pro-inflammatory cytokines, chemokines to actuate hemodynamic compromise in CRS type I patients. Pro-inflammatory cytokines not only aggravate fluid retention and venous congestion but also initiate apoptosis and oxidative stress. The immune response's primary motive is to elicit the heart and kidney to produce cytokines, intensifying the inflammatory process. Despite the possible standard of care, patient mortality, treatment cost, readmissions are extreme in CRS type I, and inflammation certainly has critical inferences warranting future research in humans.

## Introduction and background

“Physiological interaction between organs is necessary for the optimal equilibrium and functioning of the organism” [1]. The human body continuously strives to achieve one critical goal: homeostasis. The human body has an intricate communication system, collectively termed as “organ cross-talk.” Irrespective of their distance, the synergy between each organ, tissue, and cell is vital to attain equilibrium and a stable internal environment. This communication is in the form of hormones, proteins, mechanical stimuli, and electrical signals. Altering this cross-talk produces some drastic consequences, adaptive or maladaptive. Cardiorenal syndromes are such instances where specific maladaptive changes alter the bidirectional link between heart and kidneys. The term cardiorenal syndromes constitute of five subtypes: in type I (acute cardiorenal syndrome), rapid cardiac dysfunction causes acute kidney injury (AKI) while in type II (chronic cardiorenal syndrome), chronic cardiac disorders invoke chronic kidney disease. On the same spectrum, in type III (acute nephrocardiac syndrome) and type IV (chronic nephrocardiac syndrome), AKI and chronic kidney disease surmount to acute heart failure. In type V, nonetheless, a systemic illness elicits simultaneous damage of heart and kidneys [2]. This article focuses on cardiorenal syndrome type I (CRS type I). Etiology of CRS type 1 stems from sudden and abrupt pump failure due to conditions such as cardiogenic shock, acute decompensated heart failure (ADHF), acute coronary syndrome, and cardiac surgery [2-4]. In patients with acute decompensated heart failure and acute heart failure, CRS type 1 represents an astonishing 25% to 33% of patients and 27% to 45% of patients in the hospital, respectively [5-9]. Most patients first manifest laboratory evidence of AKI - an increase in serum creatinine greater than 0.3 mg/dL - and gradually reveal symptoms of kidney dysfunction three to five days after admission [10-12]. Although AKI is an independent predictor of mortality, it also has significant implications for prognosis, treatment cost, hospitalization duration, readmissions, cardiovascular events, and rapid progression to chronic kidney disease in CRS type I patients [12-14]. As the disease evolves, underlying pathology holds the key to unravel promising therapeutic and diagnostic benefits. Like its counterparts, the pathophysiology of CRS type I is multifactorial. There are hemodynamic mechanisms - arterial underfilling, decreased cardiac output, increased venous congestion, hyponatremia, and non-hemodynamic mechanisms - activation of the sympathetic nervous system and renin-angiotensin-aldosterone-system (RAAS) including the uncontrolled formation of reactive oxygen species (ROS) and nitric oxide [15-17]. One of the most speculated non-hemodynamic mechanisms of CRS type I is the immune system. The immune response is a powerful defense system adapted by the human body that has drastic repercussions if gone rogue. A savior and savage, immune system orchestrates organ healing and damage mediated by many components [18]. The normal immune response occurs with antigen recognition/presentation, activation of the complement and innate/ adaptive immune systems, and finally, resolution of the response. Interestingly, studies have shown that the function of the immune system is skewed in CRS type I partly because of improper inflammatory stimulation and inhibition [4]. Recent evidence shows that patients with severe heart failure and AKI demonstrate high pro-inflammatory cytokines, chemokine up-regulation, neutrophil migration and extravasation, toll-like receptor expression, and unregulated apoptosis leading to oxidative stress [4,19]. In CRS type I, the immune response induces a high-pressure system, fabricates mechanical stress in the vasculature, and promotes additional inflammation [20]. Numerous researches are underway to elaborate on the influence of the immune system in CRS type I, but the disease’s complex nature poses a significant limitation. Recent studies analyzed the overzealous immune system in CRS type I, and how it evokes multiple organ damage. Nevertheless, a few crucial aspects remain in question: Does the immune system govern other etiologic factors in CRS type I? What triggers the immune system in CRS type I? Is immune response the culprit for patient deterioration and high mortality? Will there be any therapeutic benefit from immunomodulation? This review primarily centers on immune system dominance over CRS type I pathogenesis. 

## Review

Discussion

**Figure 1:**
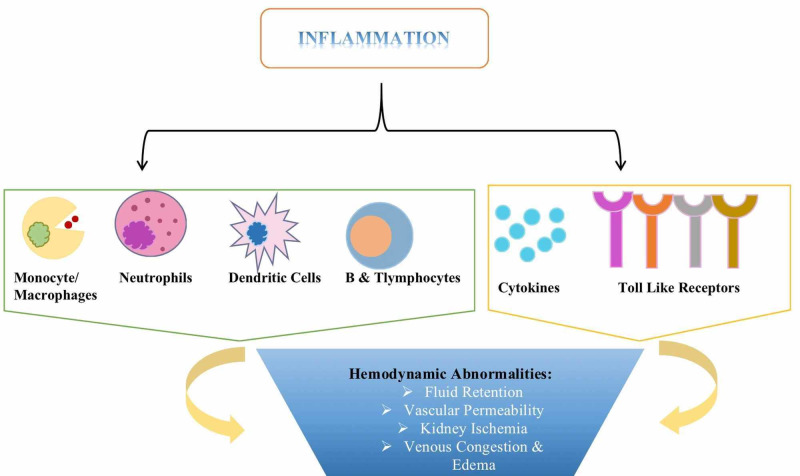
Various inflammatory components instrumental in the hemodynamic pathophysiology of cardiorenal syndrome (CRS) type I

CRS type I is characterized by a multifactorial etiology; however, there is a causal relationship between the factors that promote functional deterioration of the heart and kidney. Changes at the cellular level are fundamental for detrimental tissue remodeling and aberrant organ cross-talk. As shown in Figure 1, inflammation instigates an environment to advance these microscopic changes through its integral units- cells, cytokines, and toll-like receptors (TLRs) [4]. These components mediate the hemodynamic abnormalities of CRS type I. Fluid reabsorption is a strictly controlled process in the pulmonary interstitium, and immune activation interferes with the reabsorption process causing fluid overload in the lungs [21,22]. Inflammation creates a framework for third spacing that contributes to many of the hemodynamic abnormalities in the kidney. A few of the underlying mechanisms are insufficient renal perfusion with a subsequent decrease in glomerular filtration rate (GFR), ischemic damage to the tubular epithelium, and peritubular edema [4]. 

Inflammation superimposes itself on CRS type I and plays a pivotal role in the pathophysiology of the disease. There are four forms of CRS: in type I and II, recent cardiac injury incites AKI and chronic kidney disease. However, in types III and IV, acute exacerbation of chronic cardiac disease promotes AKI and the sudden worsening of chronic kidney disease [4]. Excluding the first subtype, the remaining three subtypes are acute on chronic decompensation of heart or kidney disease. A noteworthy characteristic of chronic diseases is a subtle yet concealed and active immune response. Hence, when CRS type I manifests symptomatically, a formerly well established inflammatory activation gets magnified and sets in stone a series of events, including hemodynamic consequences that increase mortality in these patients. 

Innate Immune System

**Figure 2:**
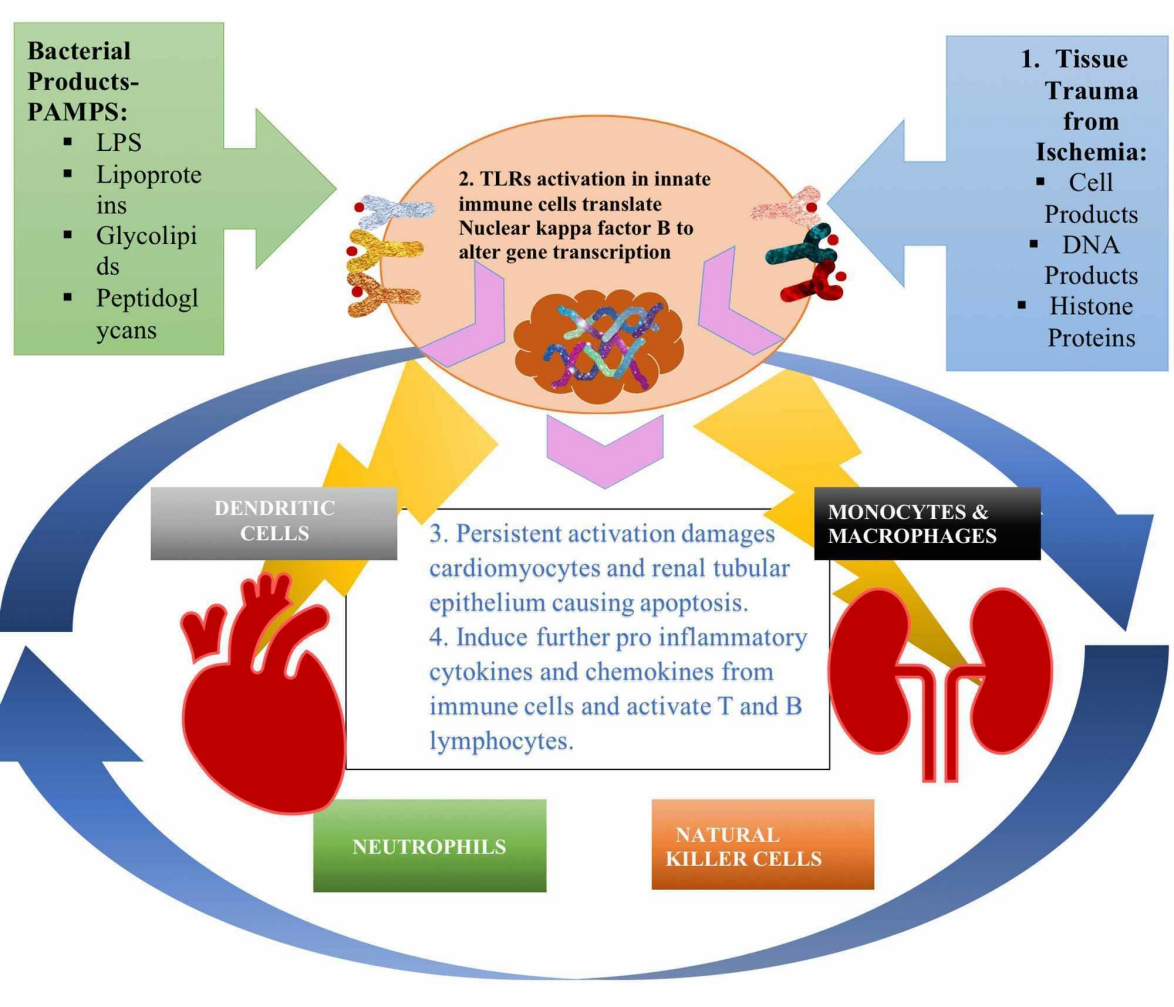
Mechanisms that activate toll-like receptors (TLRs) and their impact in cardiorenal syndrome (CRS) type I pathophysiology. 1. Tissue trauma releases products called endogenous ligands that stimulate TLRs. This pathologic trigger is the earliest immune response in AKI injury that sets a cascade of events in motion, which eventually causes massive tissue damage, 2. Amplification of pro-inflammatory cytokines occurs through translocation of transcription factor nuclear factor-kappa B to the nucleus, 3. Sustained TLR activation and inflammatory response leads to apoptosis of renal tubular epithelium, 4. TLRs fuel further cytokine release from neutrophils, monocyte/macrophages, neutrophils, and natural killer cells, resulting in the adaptive immune system activation.

The first line of defense from foreign antigens is the innate immune system. Cells of the innate immune system are perpetually circulating in the blood monitoring for any signs of insults in the organ systems. Nevertheless, during chronic illnesses, tissue injury releases ROS and reactive nitrogen species (RNS) along with mitochondrial products provoking the inflammatory response [5]. In terms of CRS type I and as Figure 2 illustrates, neutrophils, dendritic cells (DCs), monocytes/macrophages are integral to the dysfunctional immune response. Notably, after an ischemia-reperfusion injury, neutrophils infiltrate tissues, produce harmful proteases, ROS, and myeloperoxidase (MPO) enzyme debilitating organ function [23,24]. Table 1 reveals numerous studies on components of the innate immune system and their interpretation of CRS type I pathology. 

**Table 1:** Studies detailing the effects of the innate immune system in cardiorenal syndrome (CRS) type I. ACE- angiotensin-converting enzyme, AKI- acute kidney injury, IL- interleukin, TLR- toll-like receptors, TNF- tumor necrosis factor, GM-CSF- granulocyte/monocyte-colony stimulating factor, G-CSF- granulocyte-colony stimulating factor.

AUTHOR & YEAR OF PUBLICATION	TYPE OF STUDY	PURPOSE OF STUDY	CONCLUSION
Clementi et al. [1] 2019	Review	Neurohormonal, endocrine, immune dysregulation and inflammation in CRS	To insinuate the need for novel drug therapies that cover new mechanisms in CRS.
Virzì et al. [5] 2014	Review	The Hemodynamic and Non-hemodynamic Cross-talk in CRS type I	To elaborate molecular, cellular, and subcellular features for advancing treatments
Virzì et al. [18] 2014	Review	Heart--kidney cross-talk and role of humoral signaling in critical illness	Damaged cardiac myocytes and renal tubular epithelium promote activation of innate and adaptive immune systems strengthening the humoral response.
Pasare and Medzhitov [25] 2004	Review	Toll-like receptors: linking innate and adaptive immunity	TLRs are essential for immune cell maturation and the apt recognition of internal and external molecular pathogens
de kleijn and Pasterkamp [26] 2003	Review	Toll-like receptors in cardiovascular diseases	TLRs play a crucial role in initiating cardiovascular diseases especially atherosclerosis formation
Chao [27] 2009	Review	Toll-like receptor signaling: a critical modulator of cell survival and ischemic injury in the heart	TLRs respond to tissue injury and are important sources of cardiac ischemia-reperfusion injury. These receptors also cardioprotectors regulating cell survival in heart.
Eissler et al. [28] 2011	Animal study	Hypertension augments cardiac Toll-like receptor-4 expressions and activity	Hypertension actives the innate immune system through TLR. ACE inhibitors inhibit inflammation only at high doses
Allam et al. [29] 2012	In vitro study	Histones from dying renal cells aggravate kidney injury via TLR2 and TLR4	Histone neutralization can reduce ischemia-reperfusion injury from dying renal epithelial cells.
Kinsey and Okusa [30] 2008	Review	Inflammation in Acute Kidney Injury	Ischemia-reperfusion injury stimulates the immune system. TLR, chemokines. Cytokines amplify this response. Further research is needed to categorize the function of each component of the immune system in AKI.
Dong et al. [31] 2007	Animal study	Resident dendritic cells are the predominant TNF-secreting cell in early renal ischemia-reperfusion injury	Renal dendritic cells are localized to renal peritubular space and respond to innate immunity enrolling further circulating immune cells into the kidney.
Virzì et al. [32] 2018	Review	The role of dendritic and endothelial cells in CRS	Heart and kidney dendritic cells are involved in tissue remodeling. Additionally, endothelial cells act as antigen-presenting cells and act as a bridge between innate and adaptive immune systems.
Zhang et al. [33] 1993	Animal study	Interstitial dendritic cells of the rat heart. Quantitative and ultrastructural changes in experimental myocardial infarction	These dendritic cells instigate additional lymphocytes and reduce in quantity with the formation of a scar.
Naito et al. [34] 2008	Animal study	Differential effects of GM-CSF and G-CSF on the infiltration of dendritic cells during early left ventricular remodeling after myocardial infarction	Induction of MI in rat models is characterized by dendritic cells infiltration, increased interferon-gamma and TLR4 expression with decreased IL-10 levels
Vaduganathan et al. [35] 2013	Review	The immunological axis in heart failure: the importance of the leukocyte differential	High lymphocyte counts in acute and chronic heart failure patients are associated with unfavorable prognosis. In particular, elevated monocyte counts are also indicative of severe outcomes in heart failure
Wrigley et al. [36] 2011	Review	The role of monocytes and inflammation in the pathophysiology of heart failure	Persistent activation of monocytes during heart failure augments tissue injury and are implicated in disease progression
Satoh et al. [37] 2008	Review	Immune modulation: role of the inflammatory cytokine cascade in the failing human heart.	Cytokines and TNF-alpha induce changes in monocyte phenotype, myocardial cell death, and advanced matrix metalloproteinase activity to enhance ventricular remodeling
Pastori et al. [38] 2015	In vitro study 40 patients	CRS type I: A Defective Regulation of Monocyte Apoptosis Induced by Pro-inflammatory and Proapoptotic Factors	Inflammatory cytokines in the plasma of CRS patients induce the production of more cytokines leading to unregulated apoptosis.

TLRs induce activation of the innate immune response. They are transmembrane proteins located on the plasma membrane of leukocytes and lymphocytes cardinal for mounting an immune response. Ten subtypes of TLRs dimerize with each other to recognize pathogen-associated molecular patterns (PAMPs) traversing through our bodies, depicted in Figure 2. These receptors create a functional change in the immune cells activating them to secrete cytokines (IL-1 beta, IL-6, and tumor necrosis factor (TNF)-alpha) and chemokines (keratinocyte chemoattractant-1 and monocyte chemoattractant protein-1) [25]. During CRS type I and especially AKI, TLRs play a critical role in influencing inflammatory activation and poor bidirectional communication. Studies have shown that TLRs are involved in various cardiac pathologies, such as acute myocardial infarction, cardiac failure, ischemic myocardial injury, and myocardial dysfunction [26-28]. 

Virzì et al. discuss the aftermath in AKI after inducing the TLR pathway; Figure 2 reveals the critical events related to TLRs activation [18]. Similarly, Clementi and colleagues mentioned that TLRs incite innate and adaptive immune systems, coordinating the deleterious cardiorenal cross-talk [1]. Allam et al. defined another perspective for TLR signaling where histones released from dying tubular epithelium initiate cytokine production and exacerbate kidney injury [29]. These studies suggest that TLRs are essential components of immunity and serve as bridges between innate and adaptive immune systems. Furthermore, inflammation is one of the earliest responses to tissue injury that damages the bidirectional communication in CRS type I and sets the platform for ensuing pathogenesis. 

DCs are antigen-presenting cells (APC) of the innate immune system that provoke adaptive immune cells. As noted in Figure 2, they depend on TLRs for maturation and always scrutinize for antigens in many tissues. These cells interact with other subtypes using CD40-CD40 ligand and produce many pro-inflammatory substances, including interleukin (IL)-12, IL-6, TNF-alpha, and monocyte chemoattractant protein-1 [30,31]. Clementi et al. elucidated the vital role of renal dendritic cells in the inflammatory response and, after maturation, increase MHC II expression levels and costimulatory molecules [1]. Additionally, Virzì et al. emphasized that DCs maturation and antigen presentation regulates CD4+ and CD8+ T lymphocyte function and proliferation [5]. In other articles, however, Virzì and colleagues also shed light on the unclear contribution of DCs in AKI, including their part in the pathophysiology of CRS type I [18,32]. The majority of the data regarding DCs in heart and kidney illnesses arises from murine models where research-induced ischemic- reperfusion injury mimics in vivo effects [33,34]. 

Virzì and Clementi extrapolated their ideas about the role of dendritic cells in CRS type I from mouse models. Since these researchers chronicled this emerging evidence of DCs in CRS type I, there has been no further research or clinical trials to document the effects of DCs in CRS dysfunction. Although animal studies cannot be applied to humans precisely, these studies prove that inflammatory cells are principal for the dysfunction associated with cardiorenal syndromes, warranting future research in patients. 

The monocyte/macrophage system is another potent immune regulator. They produce cytokines that enhance the immune response and function as APCs to the B and T lymphocytes. Notably, in heart failure, monocytes are the primary cells for cytokine generation and are also responsible for most of the inflammatory process in heart diseases [35-37]. Concerning CRS type I, there are dual points of observation for monocytes. Virzì et al., in their review, acknowledged the presence of monocytes/macrophages in myocardial pathologies; however, they also asserted the uncertain contribution of these cells in repair or damage of the myocardium [5].

Additionally, while the in vitro study led by Pastori et al. supported the pro-inflammatory effect of monocytes, they also endorsed the theory of monocyte apoptosis from cytokine laced plasma of CRS type I patients [38]. Specifically, Clementi et al. also stressed the significance of high inflammatory levels associated with acute heart failure caused monocytes’ death and oxidative stress [1]. Even though in vitro studies are the weakest evidence, they laid the groundwork to enunciate the vicious milieu created by the immune system’s components. 

Provocation of Humoral Signaling and Its Repercussions

This moiety of the immune system includes water-soluble mediators that principally strive to reinforce initial immune response. Standard humoral signaling components include cytokines, chemokines, interferons, and growth factors. Cytokines such as interleukins establish powerful communication strategies between white blood cells while chemokines facilitate chemotaxis, especially for neutrophils, and interferons have antiviral properties [5]. The continuous presence of these mediators magnifies immune response, precipitates inter/intracellular changes, deteriorates organ function, and impairs organ cross-talk [5]. Table 2 enumerates on recent studies, detailing humoral signaling function in CRS type I. 

**Table 2:** Studies elucidating the effects of humoral signaling on cardiorenal syndrome (CRS) type I pathogenesis IL- interleukin

AUTHOR & YEAR OF PUBLICATION	TYPE OF STUDY & # OF PATIENTS	PURPOSE OF STUDY	CONCLUSION
Clementi et al. [1] 2019	Review	Neurohormonal, endocrine, immune dysregulation and inflammation mechanisms in CRS	To insinuate the need for novel drug therapies that cover new mechanisms in CRS.
Ronco et al. [4] 2012	Review	CRS type I: Pathophysiological Crosstalk Leading to Combined Heart and Kidney Dysfunction in Acutely Decompensated Heart Failure	Multifactorial mechanisms lead to progressive heart and kidney dysfunction. New diagnostic and therapeutic strategies decrease the morbidity associated with CRS type I.
Virzì et al. [5] 2012	Review	The Hemodynamic and Non-hemodynamic Cross-talk in CRS type I	To elaborate molecular, cellular, and sub-cellular features for advancing treatments.
Virzì et al. [18] 2014	Review	Heart--kidney cross-talk and role of humoral signaling in critical illness	Damaged cardiac myocytes and renal tubular epithelium promote activation of innate and adaptive immune systems strengthening the humoral response.
Colombo et al. [20] 2012	Review	Inflammatory activation: cardiac, renal, and cardio-renal interactions in patients with cardiorenal syndrome	To highlight the sustained inflammatory response that is responsible for the functional deterioration of patients with CRS. Existing anti-inflammatory treatment methods have been disappointing to date necessitating new studies.
Pastori et al. [38] 2015	In vitro study, 40 patients	CRS type I: A Defective Regulation of Monocyte Apoptosis Induced by Pro-inflammatory and Proapoptotic Factors	Inflammatory cytokines (IL-6, IL-18) in the plasma of CRS patients induce the production of more cytokines leading to unregulated apoptosis.
Virzì et al. [39] 2018	In vitro study, 53 patients	Levels of Pro-inflammatory cytokines, oxidative stress, and tissue damage markers in patients with acute heart failure with and without CRS type I	High levels of Pro-inflammatory cytokines, oxidative stress, and biomarkers are the crux of CRS type I pathophysiology.
Virzì et al. [40] 2012	In vitro study, 15 patients	CRS type I may be Immunologically Mediated: A Pilot Evaluation of Monocyte Apoptosis	Inflammation is the basis for organ damage and impaired apoptosis in CRS type I patients. CRS type I patients plasma can trigger apoptosis in monocytes.
Pastori et al. [41] 2015	In vitro studies, 29 patients	CRS type I: Activation of Dual Apoptotic Pathways	Inflammation induced apoptosis of renal tubular epithelial cells, which is a fundamental pathogenic mechanism in CRS type I and a potential future therapeutic target.

**Figure 3:**
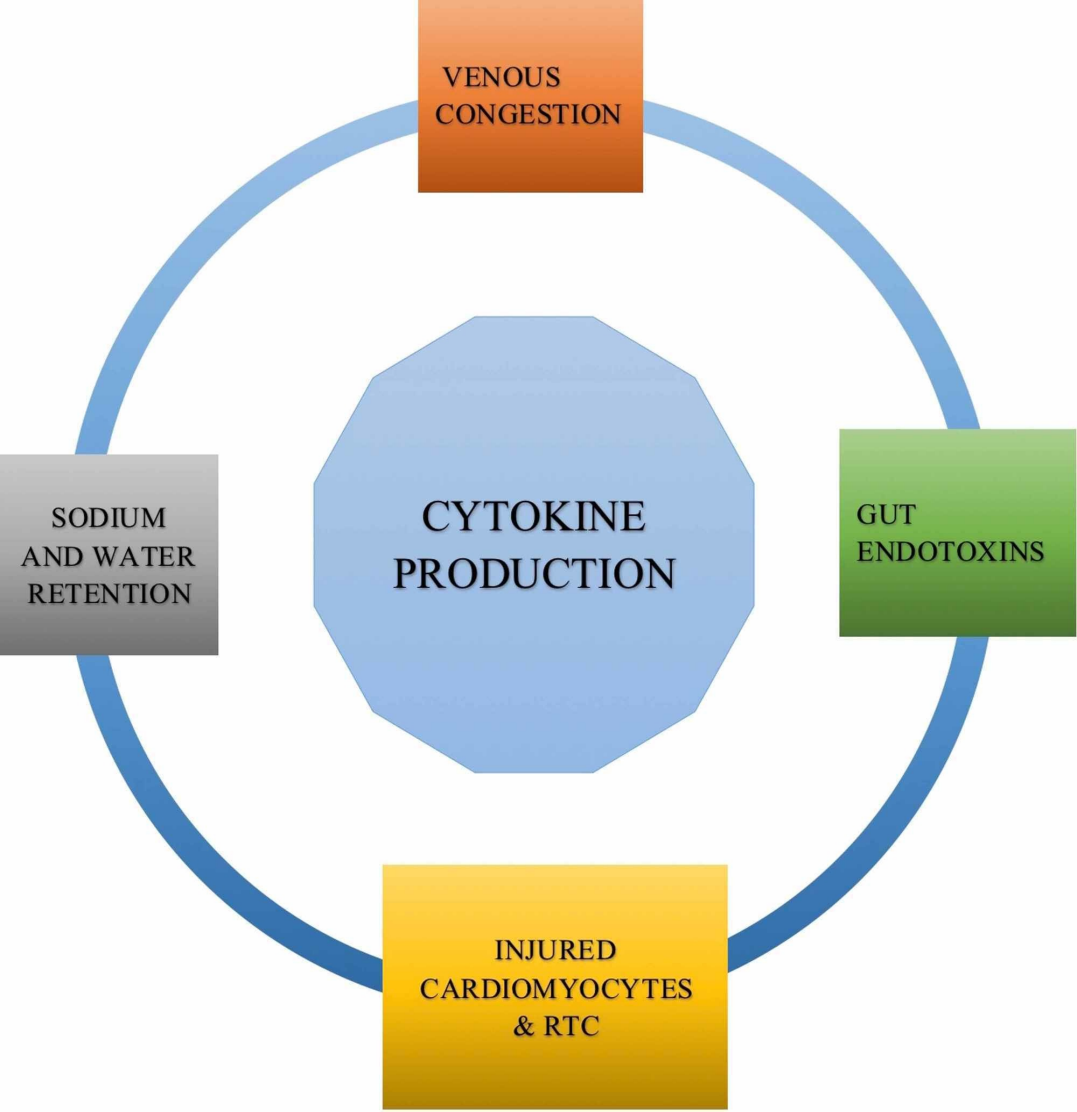
Sources of cytokine production in cardiorenal syndrome (CRS) type I.

Various pathological aspects in CRS type I govern cytokine production, as depicted in Figure 3. Virzì et al. stressed that the release of humoral factors from damaged cells supervises TLRs activation and increases cytokine generation- TNF-alpha, IL-1, IL-4, IL-6, IL-13, and IL-17, which are fatal in cardiac critical care units and instigate organ damage in CRS type I [18]. At the same time, Clementi et al. discussed that venous congestion in CRS type I fosters cytokine release, crippling renal function [1]. Furthermore, Colombo et al. hinted at the prospect of sodium and water retention, which aggravates fluid overload, congestion, and inflammatory cytokine production [20]. 

Independently, Ronco et al. and Colombo et al. put forward another exciting aspect- gut endotoxins induce cytokines in CRS type I [4,20]. Colombo et al. proposed that fluid overload in CRS causes bowel edema which in turn destroys intestinal villi endothelial cells, releasing lipopolysaccharide (LPS) from gut bacteria [20]. Ronco et al. elaborated on this idea and ascertained that low intestinal perfusion in CRS type I and heart failure liberates LPS promoting IL-1, IL-6, and TNF-alpha generation [4]. These reviews validate the ideology where multiple pathways exist for inflammation to debilitate organ function and impair the physiological equilibrium. Therefore, targeting one mechanism or organ system does not compensate for the systemic imbalance. 

**Figure 4:**
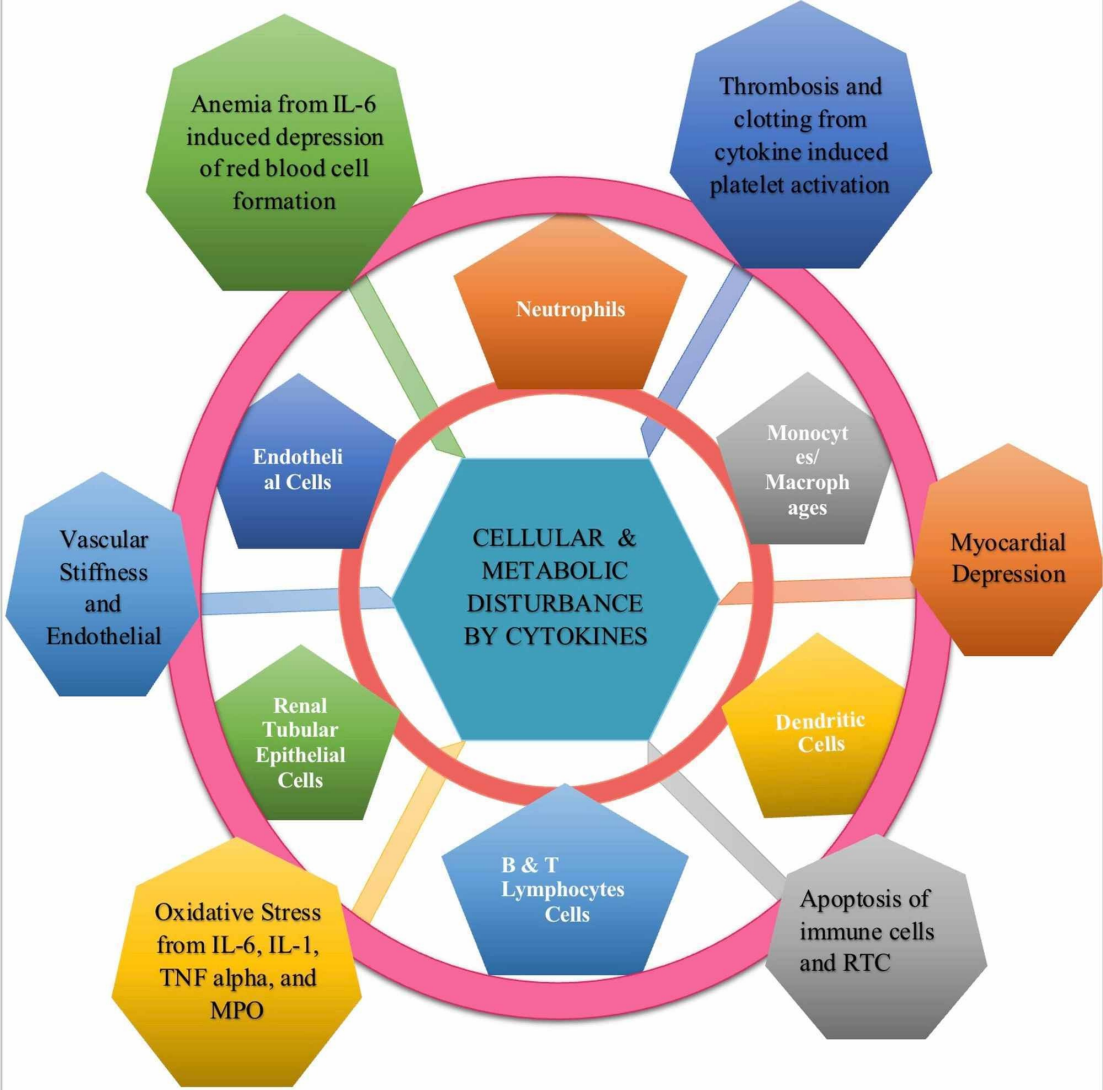
Heterogenous sequela of cytokines on different tissues and on homeostasis. MPO- myeloperoxidase, IL- interleukin, TNF- tumor necrosis factor, RTC- renal tubular cells

Humoral mediation proves that inflammation not only disturbs communication between organs but also influences metabolism in CRS type I. Figure 4 demonstrates cytokine impact on multiple cells and on homeostasis. Clementi and colleagues described the inverse relation between IL-6 and hemoglobin; pro-inflammatory cytokines cause anemia by bone marrow suppression, erythropoietin receptor down-regulation, red blood cell precursors destruction, and iron sequestration [1]. Additionally, they noted the interrelation between clotting and inflammation; immune response damages renal vessels and activates platelets, resulting in additional cytokine and chemokine production [1]. In another study, Colombo et al. explained the cardio depressant actions of TNF-alpha through increased nitric oxide [20]. Ronco and colleagues emphasized this concept of cytokine and TLRs’ role in diminishing myocardial excitability, altering resting membrane potential, modulating substrate metabolism, and responding to the sympathetic nervous system [4]. Moreover, Colombo et al. delineated the vascular abnormalities of inflammation, such as immune system induced arterial stiffness and endothelial permeability [20]. All these reviews remark on various neurohormonal effects of inflammation, and therefore, it is the principal element mediating the pathogenesis of CRS type I. 

Apoptosis is a highly regulated, essential physiologic process. Cell death is dependent on timing, and just like inflammation, it can create havoc if stimulated inappropriately. Two apoptosis pathways are intrinsic pathways that result from disturbance to intracellular homeostasis, and the extrinsic pathway results from the coupling of death receptors on the plasma membrane [18]. Virzì et al. deciphered that TLR stimulation instigates caspases to initiate pathological apoptosis of renal tubular epithelial cells [18]. In another article, Virzì and colleagues affirmed that low perfusion and hypoxia evoked cellular metabolism changes, ultimately causing cell death [39]. 

Pro-inflammatory cytokines have a dual purpose where they provoke other immune cells to produce additional inflammatory markers, creating an environment for cell death and altering organ fate. Virzì et al. incubated monocytes with CRS type I plasma and identified a two-fold increase in caspase-8 levels, a three-fold surge in IL-6, and a ten-fold hike in TNF-alpha levels [40]. Likewise, Pastori et al. analyzed this theory in their in vitro study, where they incubated monocytes and renal tubular epithelial cells in CRS type I plasma. They found high IL-6, IL-18, and TNF-alpha levels that increased humoral mediators and caspase 8 and 3 levels in monocytes, and also discerned that CRS type I plasma initiated dual apoptotic pathways [38,41]. 

In vitro studies justify the notion that inflammation alters organ destiny compared to other compensatory mechanisms. These studies also create a compelling foundation for future observational studies or clinical trials targeting the immune system as a treatment strategy. 

Oxidative Stress

Oxidative stress and inflammation are intertwined, and their clinical ramifications are a subject of interest in the medical literature. A delicate equilibrium exists between essential and excessive amounts of an element. Notably, every tissue needs a certain amount of oxidative species to protect itself, while excessive amounts can cause dysfunction, injury, and organ failure [5,42]. An ongoing inflammatory process in CRS type I renders the heart and kidney incapable of controlling oxidative stress [5]. Similar to multiple humoral signaling pathways, there are different mechanisms to bring about oxidative stress in CRS type I. Table 3 elaborates on oxidative stress-induced CRS type I pathology. 

**Table 3:** Studies explicating the importance of oxidative stress in cardiorenal syndrome (CRS) type I pathophysiology. RAS- renin-angiotensin system, SNS- sympathetic nervous system, ESRD- end stage renal disease, MPO- myeloperoxidase

AUTHOR & YEAR OF PUBLICATION	TYPE OF STUDY & # OF PATIENTS	PURPOSE OF STUDY	CONCLUSION
Lullo et al. [2] 2017	Review	Update on the pathophysiology of CRS types 1-5	To elucidate the burden of pathophysiology in CRS types 1-5 on the functioning of the heart and kidney.
Virzì et al. [5] 2014	Review	The Hemodynamic and Non-hemodynamic Cross-talk in CRS type I	To elaborate molecular, cellular, and subcellular features for advancing treatments.
McCullough [13] 2011	Review	Cardiorenal syndrome: pathophysiology to prevention	Importance of catalytic iron in organ injury and other biomarkers for diagnosis, treatment, and prognosis.
Bongartz et al. [16] 2004	Review	The severe Cardiorenal syndrome: Guyton Revisited	To unravel the harmful consequences of RAS, RNS &ROS, inflammation, and SNS.
Virzì et al. [39] 2018	In vitro study, 53 patients	Levels of Pro-inflammatory cytokines, oxidative stress, and tissue damage markers in patients with acute heart failure with and without CRS type I	High levels of Pro-inflammatory cytokines, oxidative stress, and biomarkers are the crux of CRS type I pathophysiology.
Virzì et al. [42] 2015	In vitro study, 23 patients	Oxidative stress: Dual Pathway Induction in CRS Type I Pathogenesis	To understand the imbalances of reactive oxygen species and nitrogen species in CRS type I and their implications in activating the inflammatory cascade.
Maruyama et al. [43] 2004	Review	Inflammation and oxidative stress in ESRD-the role of myeloperoxidase	Inflammation increases cardiovascular risk in end-stage renal disease. Furthermore, MPO associated oxidative stress is a significant risk factor for vascular dysfunction.

Lullo and companions drew attention to significant elevations in ROS, RNS, IL-6 levels, and MPO, which generates ROS through hydrogen peroxide and nitrogen dioxide [2,42,43]. This hypothesis was endorsed by Virzì et al. when they observed higher MPO levels, an enzyme present in neutrophil granules, in CRS type I plasma [39]. Their findings also confirmed a strong correlation between oxidative stress, immune response, IL-6, and IL-18 [39]. 

Virzì et al. [42], organized another in vitro study and presented five fundamental points on oxidative stress: 1. There was a dramatic rise in ROS and RNS species in CRS type I plasma compared to acute heart failure patients, 2. Hydrogen peroxide and nitric oxide augment IL-6 levels in CRS type I patients, 3. In CRS type I, inflammation reduces the metabolism of free fatty acids decreasing myocardial ATP levels, increasing glycolysis, and enhancing ROS, 4. ROS disturbs cardiomyocyte contractility, calcium processing, and ion transport affecting cardiac and renal function, 5. Diabetes was a strong risk factor for oxidative stress as hyperglycemia substitutes other pathways for glucose metabolism. 

Furthermore, Bongartz stressed that oxidative stress provokes IL-6, IL-1, and TNF-alpha, and hinders renal compensatory mechanisms [16]. Another take on ROS generation is iron, and McCullough put forward this initiative in his review. He rationalized that unstable iron converts into hydroxyl radicals, which result in cellular dysfunction and apoptosis [13]. All these studies on oxidative stress corroborate the theory inflammation has a spectrum of molecular manifestations that depress heart and kidney function in CRS type I. These disturbances clinically deteriorate patients making them refractory to treatment. 

Biomarkers

In chronic diseases, biomarkers are crucial elements. Their rising levels indicate occult pathologies before apparent evidence of symptoms. In CRS, these markers indicate heart and renal dysfunction and anticipate renal injury in cardiac disease [44]. Additionally, they predict prognosis, assess treatment efficacy, and aid in treatment strategy [44]. Inflammatory biomarkers are the earliest markers to spike; however, clinicians do not prefer to utilize them. Table 4 mentions studies about the diagnostic purpose of inflammatory biomarkers in CRS type I. 

**Table 4:** Studies manifesting evidence of biomarker contribution in cardiorenal syndrome (CRS) type I diagnosis.

AUTHOR & YEAR OF PUBLICATION	TYPE OF STUDY & # OF PATIENTS	PURPOSE OF STUDY	CONCLUSION
Ronco et al. [6] 2009	Review	Cardio-Renal Syndromes: a report from the consensus of the Acute Dialysis Quality Initiative	To define the epidemiology, biomarkers, preventive strategies that can direct future research, including clinical trials.
McCullough [13] 2011	Review	Cardiorenal syndrome: pathophysiology to prevention	Importance of catalytic iron in organ injury and other biomarkers for diagnosis, treatment, and prognosis.
Virzì et al. [39] 2018	In vitro study, 53 patients	Levels of Pro-inflammatory cytokines, oxidative stress, and tissue damage markers in patients with acute heart failure with and without CRS type I	High levels of Pro-inflammatory (IL-6, IL-18) cytokines, oxidative stress, and biomarkers are the crux of CRS type I pathophysiology.
Fu et al. [44] 2018	Review	Biomarkers in Cardiorenal Syndromes	To identify the importance of various biomarkers in renal injury caused by cardiac dysfunction.

**Figure 5:**
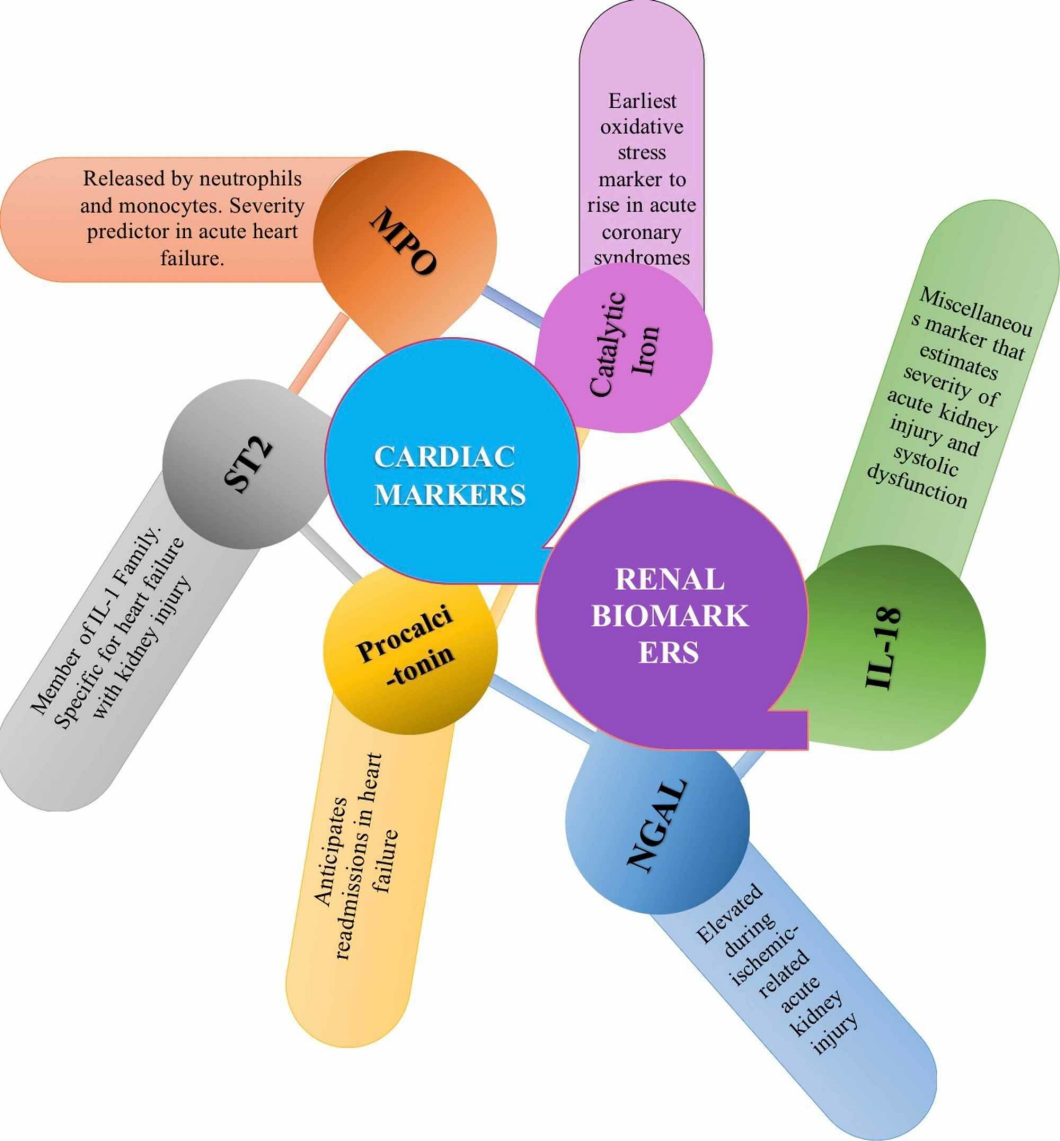
Inflammatory cardiac and renal biomarkers during acute heart failure and acute kidney injury (AKI). MPO- myeloperoxidase, NGAL- neutrophil gelatinase-associated lipocalin, IL- interleukin, ST2- suppression of tumorigenicity 2

Fu et al. [44], enlightened biomarkers function in their review. They focused on inflammatory and non-inflammatory markers, but for this review, we shall solely enumerate on inflammatory markers in CRS, as seen in Figure 5. Cardiac inflammatory markers include: 1. MPO- released by neutrophils and monocytes, predicts disease severity in acute heart failure and kidney disease, 2. Suppression of tumorigenicity 2 (ST2)- a member of the IL-1 family, is specific for the prognosis of heart failure with kidney malfunction, 3. Procalcitonin- usually elevated after bacterial infection, but also assess for readmissions in heart failure. Renal inflammatory biomarkers include: 1. Neutrophil gelatinase-associated lipocalin (NGAL)- increases in ischemic AKI and anticipates worsening renal failure, 2. IL-18- a marker of blood vessel stiffness that exacerbates kidney injury and foresees mortality in systolic dysfunction. Figure 5 represents an overview of cardiac and renal inflammatory markers. 

Moreover, McCullough elaborated on NGAL, IL-18, and catalytic iron in CRS [13]. As Figure 5 also demonstrates, catalytic iron is the root of the lethal hydroxyl radical and rises before serum troponin in acute coronary syndromes. NGAL or siderocalin, the first renal marker to be detected in blood and urine after AKI, is a scavenger of catalytic iron limiting oxidative stress. IL-18 increases 48 hours before creatinine and has an area under the operating curve of >90% for sensitivity and specificity in ischemic AKI. Virzì and assistants in their in vitro study emphasized the importance of high NGAL levels in CRS type I patients on the third day of admission [39]. Similarly, Ronco et al. prioritized NGAL because one urinary NGAL measurement in AKI patients renders a sensitivity of 90% and specificity of 99% [6].

In the medical literature, most inflammatory markers like C-reactive protein (CRP) or erythrocyte sedimentation rate (ESR) are non-specific markers of inflammation in chronic diseases. Nonetheless, these studies stress on inflammatory biomarkers that specifically pertain to cardiac or renal damage. Compared to non-inflammatory markers, NGAL and IL-18’s statistics validate their accuracy in CRS.

Non-Immune Cells With Inflammatory Profile

In CRS type I, two cell lines succumb to immune attack and gradually change their course: endothelial cells (EC) and renal tubular epithelium. Endothelial cells are the medium between blood and tissues controlling vascular, inflammatory, and coagulation stability [32]. Vascular dysfunction emanates with endothelial remodeling that affects their function and the organs they serve. On the other hand, renal tubular epithelium, delicate tissue of the kidney, has different coping mechanisms that feed-forward into the inflammatory response and subsequently loses its structure and function [45]. Table 5 documents evidence on the perpetual vicious circle created by inflammation using endothelium and renal tubular epithelium. 

**Table 5:** Studies validating additional immune responses from the endothelium and renal tubular epithelium in cardiorenal syndrome (CRS) type I.

AUTHOR & YEAR OF PUBLICATION	TYPE OF STUDY & # OF PATIENTS	PURPOSE OF STUDY	CONCLUSION
Clementi et al. [1] 2019	Review	Neurohormonal, endocrine, immune dysregulation and inflammation mechanisms in CRS	To insinuate the need for novel drug therapies that cover new mechanisms in CRS.
Virzì et al. [18] 2014	Review	Heart--kidney cross-talk and role of humoral signaling in critical illness	Damaged cardiac myocytes and renal tubular epithelium promote activation of innate and adaptive immune systems strengthening the humoral response.
Colombo et al. [20] 2012	Review	Inflammatory activation: cardiac, renal, and cardio-renal interactions in patients with cardiorenal syndrome	To highlight the sustained inflammatory response that is responsible for the functional deterioration of patients with CRS. Existing anti-inflammatory treatment methods have been disappointing to date necessitating new studies.
Virzì et al. [32] 2018	Review	The role of dendritic and endothelial cells in CRS	Heart and kidney Dendritic cells are involved in tissue remodeling. Additionally, endothelial cells act as antigen-presenting cells and act as a bridge between innate and adaptive immune systems.
Bonventre [45] 2003	Review	Dedifferentiation and proliferation of surviving epithelial cells in acute renal failure.	The renal epithelium can regenerate and replace dead tubular cells after ischemic-repercussion injury.
Laxmanan et al. [46] 2005	In vitro study	CD40: a mediator of pro- and anti-inflammatory signals in renal tubular epithelial cells.	CD40 serves a dual purpose, promoting inflammatory ROS and anti-inflammatory heme oxygenase-1. Finding the balance between these two effects has a therapeutic purpose in inflammatory renal disease.

Clementi and colleagues, postulated endothelial transformation effects on the heart and kidney. In AKI, endothelial injury boosts inflammation, thrombosis, and vasoconstriction while in heart failure, it influences afterload and preload [1]. Virzì et al., in their review, analyzed the cytokine-induced accumulation of MHC class I and adhesion molecules- ICAM-1, ICAM-2, VCAM-1, E-selectin, P-selectin on ECs that function as unconventional APCs [32]. Colombo and companions further clarified TNF-alpha and IL-1 beta as the primary cytokines that actuate cell adhesion molecules on endothelium and also diminish nitric oxide-mediated vasodilation [20]. Vasoconstriction compromises hemodynamics of the heart and kidneys, worsening venous congestion that stimulates endothelium to produce additional cytokines [20]. 

On the contrary, tubular epithelium alters cell surface molecules and stimulatory pathways to withstand inflammatory stress. As noted by Virzì et al., endothelial permeability opens the gate for immune cell invasion into the renal interstitium, causing epigenetic and DNA changes [18]. As manifested by Laxmanan et al. in their in vitro study, epigenetic alterations encourage the cells to express more inflammatory receptors like CD40/CD40-ligand [46]. Inflammation recruits other cells into the immune response, thereby creating a vicious circle. These recent studies prop up the feed-forward mechanism, magnifying the anomalous type I cardiorenal cross-talk. 

We were not able to find any randomized controlled trials and observational studies relevant to this topic. Therefore, we could not establish causal relationships delineating risk factors and inflammation-related incidence and prevalence. General questions that could guide future research include: 1. Could the combination testing of inflammatory biomarkers confirm the dominance of inflammation? 2. Can these markers also be used to categorize the severity profile of CRS type I patients? 3. Does the inflammatory remodeling of cardiac and renal parenchyma guide future research to define diagnostic and therapeutic protocols? 4. Should patients with cardiac abnormalities have a regular checkup of inflammatory markers? 5. What is the specific treatment for CRS type I? 6. What are the treatment options to control the inflammatory process in CRS type I patients? 

Limitations

Unfortunately, most of the evidence in this paper comes from other reviews, animal studies, and in vitro studies from patients in Italy. There were few reviews from the United States, but the data for in vitro studies came from CRS type I patients in Italy. Pertinent data from in vitro studies is inapplicable to clinical practice due to limited patients and high bias. We formulated only mere associations about inflammatory control on CRS type I pathology. Another limitation of this paper is the lack of human studies to establish certain prognostic factors like NGAL, ST2, and IL-18 as risk factors in CRS type I patients.

## Conclusions

CRS type I challenges clinicians and complicates patient recovery. Neurohormonal mechanisms have been the interest of diagnosis and treatment in clinical care, but the disease’s mortality and morbidity are sorely high, indicating a hidden medium playing along. Inflammation governs the hemodynamics, end-organ dysfunction, and severity of CRS type I. AKI is a dreadful aspect of this disease extending hospitalization periods, cost of care, and risk of adverse events. 

A hyperactive immune system sabotages cardiorenal cross-talk and sets the ground for injurious cellular remodeling. In CRS type I, inflammation facilitates this damage through its components, altering homeostasis and metabolism, breaking barriers, and disintegrating communication. Since inflammation can modulate heart and kidneys’ identity, a swift change in the diagnostic and therapeutic approach of CRS type I is necessary. In summary, the high prevalence of this syndrome entails future human clinical trials not only to catch the disease at an appropriate time but also to discover new treatment options to curb inflammation.
